# Effective Stabilization of Organic Cathodes Through Formation of a Protective Solid Electrolyte Interface Layer via Reduction

**DOI:** 10.1002/cssc.202401599

**Published:** 2024-11-25

**Authors:** Yonglin Wang, Zhe Huang, Xiguang Gao, Razieh Fazaeli, Yuning Li

**Affiliations:** ^1^ Department of Chemical Engineering Waterloo Institute for Nanotechnology (WIN) University of Waterloo, Waterloo 200 University Avenue West Waterloo, Ontario N2 L 3G1 Canada

**Keywords:** Cathode, 2,5-dihydroxy-1,4-benzoquinone (DHBQ), Low voltage, Morphology, Solid electrolyte interphase (SEI)

## Abstract

Organic electrode materials offer a promising alternative for lithium‐ion batteries due to their lower costs, reduced environmental impact, renewability, and high theoretical capacity. Among them, 2,5‐dihydroxy‐1,4‐benzoquinone (DHBQ) is a promising cathode material, but its high solubility in electrolytes leads to rapid capacity degradation of the battery. This study investigates the dilithium salt of DHBQ, Li_2_DHBQ, as a cathode material for LIBs. Despite its minimal solubility in the electrolyte, Li_2_DHBQ cathodes suffer rapid capacity decay due to severe morphological damage within the voltage range of 1.5–3.0 V. To achieve morphological stabilization, we promoted the formation of a protective solid electrolyte interphase (SEI) layer on Li_2_DHBQ particles by lowering the discharge cutoff voltage. Cycling the battery with a 0.5 V discharge cutoff voltage achieved the optimal thickness and organic‐rich composition of the SEI layer, leading to significantly improved morphological stability of Li_2_DHBQ. Consequently, the battery maintained 170 mAh g^−1^ with a low decay rate of 0.16 % within a voltage range of 0.5–3.0 V after 200 cycles at 500 mA g^−1^. Furthermore, initial cycling with a discharge cutoff voltage of 0.5 V for 20 cycles to form an SEI layer, followed by cycling at a normal discharge cutoff voltage of 1.5 V, retained an even higher capacity of 187 mAh g^−1^ after 200 cycles at 500 mA g^−1^. These are significant improvements compared to the battery cycled only in the normal range of 1.5–3.0 V, which retained a capacity of 87 mAh g^−1^. This study demonstrates the effectiveness of forming a cathode SEI layer at low discharge voltages as a new approach to stabilizing organic cathode materials.

## Graphical abstract

This study investigates the dilithium salt of 2,5‐dihydroxy‐1,4‐benzoquinone as a cathode material for lithium‐ion batteries. Rapid capacity decay due to morphological damage is mitigated by promoting a protective SEI layer through a lower discharge cutoff voltage. Cycling at a discharge cutoff voltage of 0.5 V significantly improves battery stability.

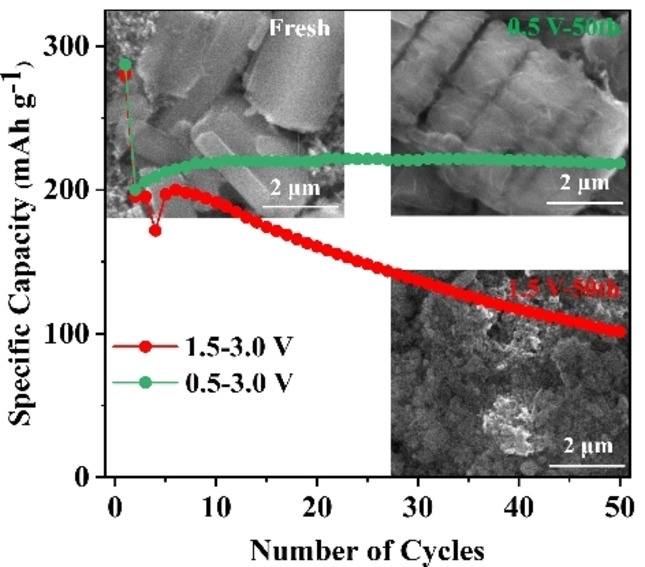

## 1 Introduction

To address climate change and the rapid depletion of fossil fuels, there has been a significant increase in demand for rechargeable batteries used in electric vehicles and electricity storage systems.[^1^, ^2^] Despite their dominance in the current rechargeable battery market, lithium‐ion batteries (LIBs) face significant challenges that limit their broader applications. High production and recycling costs, environmental pollution, and the limited capacity of existing electrode materials are among the key obstacles that need to be overcome.[^3^, ^4^, ^5^, ^6^] In response, organic electrode materials have been extensively investigated as a promising alternative, offering lower costs, reduced environmental impact, renewability, and enhanced capacity.[^7^, ^8^, ^9^] Among them, small molecule quinone compounds stand out for their high theoretical capacity and specific energy, excellent electrochemical reversibility, and natural sustainability. However, simple quinone cathode materials often exhibit high solubility in liquid electrolytes, leading to their decomposition on the anode surface and shuttling between the cathode and anode, consequently resulting in rapid capacity degradation of the battery.[^10^, ^11^] Various strategies have been explored to address the high solubility issue of some quinone cathode materials, including the utilization of mesoporous carbon,^[12]^ the synthesis of quinone‐containing polymers,^[13]^ and the formation of organo‐metal compounds.^[14]^

Among simple quinone compounds, 2,5‐dihydroxy‐1,4‐benzoquinone (DHBQ) has garnered increasing interest as a candidate electrode material due to its high theoretical specific capacity (383 mAh g^−1^), relatively high discharge potential (~2.5 V),^[15]^ and its ability to form transition metal complexes.[^14^, ^16^] Its two hydroxyl and two carbonyl functional groups readily form strong ionic and coordination bonds with divalent and multi‐valent transition metal ions, respectively, resulting in robust one‐dimensional coordination polymers (CPs)[^17^, ^18^] or three‐dimensional metal‐organic frameworks (MOFs).^[16]^ The resulting CPs and MOFs demonstrate significantly reduced solubility in electrolyte solvents,[^16^, ^18^] leading to excellent cycling stability when utilized as electrode materials compared to DHBQ.^[15]^ However, the inclusion of heavy transition metal ions compromises the theoretical specific capacity of these transition metal‐DHBQ complexes compared to their parent DHBQ.

Li_2_DHBQ has the smallest molecular weight and thus the highest specific capacity among all metal‐DHBQ compounds. While Li_2_DHBQ has been previously explored as a cathode material for LIBs, the battery exhibited rapid capacity decay, for reasons that remain unknown.^[19]^ To investigate the root cause of the poor cycling stability of Li_2_DHBQ and unlock its potential, in this study, we first measured its solubility and found that Li_2_DHBQ exhibits minimal solubility in typical battery electrolyte solvents. This indicates that factors other than solubility in the electrolyte may contribute to the rapid failure of Li_2_DHBQ batteries. Instead, SEM analysis of a Li_2_DHBQ cathode cycled within the voltage range of 1.5–3.0 V revealed severe morphological damage to Li_2_DHBQ particles, which is likely the cause of the poor cycling stability.

Our previous investigation into Cu‐DHBQ‐based LIBs highlighted the crucial role of Cu‐DHBQ′s morphological stability in battery cycling stability.^[18]^ While batteries using polyvinylidene fluoride (PVDF) as the binder degraded rapidly due to pulverization of Cu‐DHBQ particles, those employing sodium alginate (SA) exhibited remarkable cycling stability. SA was found to interact strongly with Cu‐DHBQ through polar groups, preserving its morphology and enhancing stability. These findings suggest that Li_2_DHBQ batteries may share a similar degradation mechanism involving morphological damage. However, the stabilizing effect of the SA binder on the Cu‐DHBQ cathode is limited when a typical binder concentration of 10 % is used, necessitating a large amount of 25 % SA to achieve cycling stability. This increase in binder content comes at the expense of battery energy density. Therefore, new methods are needed to protect the morphology of organic cathode materials during battery reactions and improve stability without increasing the binder content.

In LIBs, the solvent and salt in the electrolyte undergo reduction reactions at the anode, leading to the formation of an SEI layer on the anode surface.[^20^, ^21^] This SEI layer consists of a mixture of organic polymers and lithium salts with complex compositions.[^22^, ^23^] Studies have shown that the SEI layer plays a crucial role in preventing further electrolyte reactions with the anode, thereby significantly enhancing the cycling stability of LIBs.[^20^, ^24^, ^25^] Notably, the formation of SEI layers has been successfully utilized to improve the morphological stability of silicon,^[26]^ lithium metal,[^27^, ^28^] and other types of anodes.[^29^, ^30^] Similarly, an SEI layer can develop on the cathode of LIBs, also known as a cathode electrolyte interphase (CEI) layer, through oxidation reactions of the electrolyte at very high potentials during charging.[^31^, ^32^, ^33^, ^34^, ^35^, ^36^] However, its impact on cell performance is generally less pronounced compared to the SEI layer on the anode.

In this study, we developed an innovative approach to stabilize the morphology of Li_2_DHBQ and enhance cycling performance by promoting the formation of a protective SEI layer through reduction at lower discharge voltages. Our results demonstrate that the Li_2_DHBQ‐based battery with a carefully chosen 0.5 V discharge cutoff voltage exhibited greatly improved cycling stability, maintaining a high capacity of 170 mAh g^−1^ with a low decay rate of 0.16 % after 200 cycles at 500 mA g^−1^. This stability is attributed to the formation of an SEI layer with an optimal thickness and organic‐rich composition. Furthermore, initial cycling with a discharge cutoff voltage of 0.5 V for 20 cycles to form an SEI layer, followed by cycling at a normal discharge cutoff voltage of 1.5 V, retained an even higher capacity of 187 mAh g^−1^ after 200 cycles at 500 mA g^−1^. These are significant improvements compared to the battery cycled only in the normal range of 1.5–3.0 V, which retained a capacity of 87 mAh g^−1^. This study represents the first demonstration of the efficacy of forming a cathode SEI layer at low discharge voltages to stabilize organic cathode materials, offering promising potential for broader applications in other organic cathode systems.

## 2 Materials and Methods

### 2.1 Chemicals

DHBQ (98 %, Thermo Fisher Scientific), LiOH⋅H_2_O (ACS reagent, Sigma‐Aldrich), Super P (SP) (IMERYS), SA (Sigma Aldrich), N‐methyl‐2‐pyrrolidone (NMP) (anhydrous, Sigma‐Aldrich), PVDF (Mw ~1,000,000, Kynar), and other chemicals were obtained from commercial sources and used as received.

### 2.2 Synthesis of Li_2_DHBQ

To a dispersion of DHBQ (1.00 g, 7.14 mmol) in 150 mL of deionized (DI) water, a solution of LiOH⋅H_2_O (1.78 g, 7.14 mmol) in 50 mL of DI water was added while stirring at room temperature. After 2 h of reaction, a dark red solution formed. The water was removed by evaporation under reduced pressure to yield the crude product. This crude product was redissolved in ~80 ml water and rapidly added into 500 mL of acetone within 10 min to precipitate product and remove residual DHBQ and LiOH⋅H_2_O. The solid was collected by filtration, washed with acetone, and dried in a vacuum oven at 40 °C for 12 h to obtain final product Li_2_DHBQ⋅2H_2_O (hereafter referred to as Li_2_DHBQ), as a red solid (yield: 92 %). ^1^H NMR (300 MHz, D_2_O): δ 5.18 (s, 1H). ^13^C NMR (75 MHz, D_2_O): δ 100.88, 101.20, 101.52, 181.98.

### 2.3 Material Characterization

XRD measurements were performed using a Bruker D8 Discover X‐ray diffractometer equipped with Cu Kα radiation (λ=1.5418 Å). Ultraviolet–visible (UV‐Vis) spectroscopy was conducted using a Cary 7000 universal measurement spectrophotometer (UMS). SEM images were acquired using a Zeiss Sigma HD microscope. X‐ray photoelectron spectroscopy (XPS) measurements were carried out on a Thermo‐VG Scientific ESCALAB 250 microprobe with a monochromatic Al Kα X‐ray source. Transmission electron microscope (TEM) images were obtained using a Zeiss Libra 200 MC. Attenuated total reflectance Fourier transform infrared (ATR‐FTIR) spectra were measured on a Bruker VERTEX 70 FT‐IR spectrometer. ^1^H NMR and ^13^C NMR spectra were recorded on Bruker DPX 300‐MHz spectrometer in D_2_O.

### 2.4 Electrochemical Measurements

Electrochemical measurements of Li_2_DHBQ were performed using 2032 coin cells, where a lithium foil acted as the anode, Celgard 2400 served as the separator, and a 1 M lithium bis(trifluoromethanesulfonyl)imide (LiTFSI) solution dissolved in a mixture of DME and DOL (volume/volume ratio=1 : 1) served as the electrolyte. The coin cells were assembled inside an argon‐filled glovebox, with concentrations of both O_2_ and H_2_O maintained below 0.1 ppm.

To prepare Li_2_DHBQ electrode disks, a mixture of Li_2_DHBQ, SP, and SA in a weight ratio of 60 : 30 : 10 was manually ground in a mortar using a solution of DI water and ethanol (volume ratio of 9 : 1). The resulting slurry was then coated onto either aluminum (Al) or copper (Cu) foil. Each electrode had an active material loading of approximately 1 mg cm^−2^. Electrochemical performance was evaluated *via* galvanostatic measurements using a Land 2001 A battery test system. For long‐term cycling tests at a current density of 0.5 A g^−1^, the first cycle was conducted at a lower current density of 0.1 A g^−1^ to activate the cell.

## 3 Results and Discussion

### 3.1 Characterization of Li_2_DHBQ

**Figure 1:**
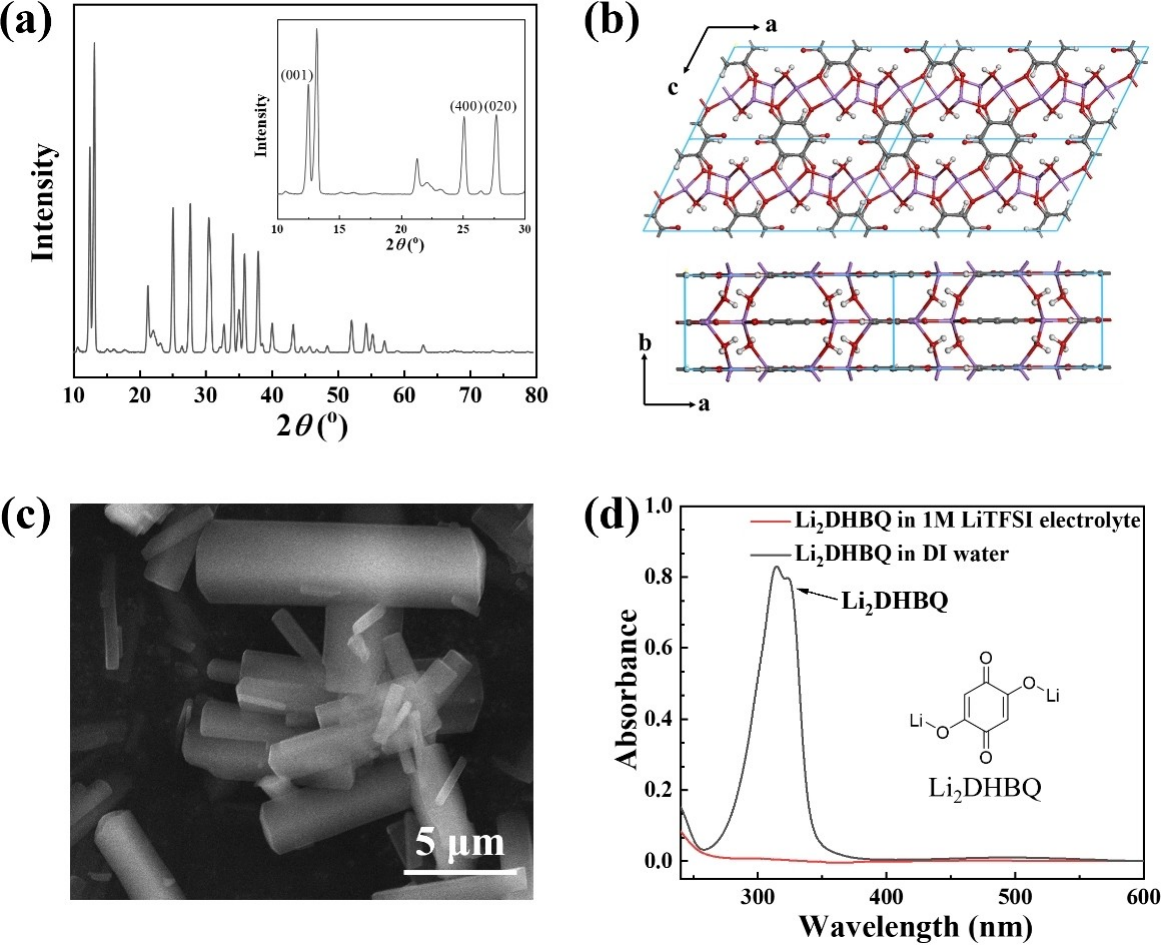
(a) Powder XRD diagram, (b) single crystal structure, (c) SEM images, and (d) UV spectra in electrolyte and DI water of Li_2_DHBQ.

The chemical and crystal structures of Li_2_DHBQ were characterized using FTIR, NMR, TGA, and XRD (Figures 1 and S1–S3; Tables S1–S2). The XRD diagram in Figure 1a exhibits a pattern consistent with previously reported data for Li_2_DHBQ, confirming a monoclinic crystal structure in the C2/m space group as illustrated in Figure 1b.[^19^, ^37^] Li_2_DHBQ units are connected via Li…O coordination bonds to form a coordination polymer. The crystallite size of Li_2_DHBQ was estimated to be around 20–30 nm using the Scherrer equation (Table S1). Given that the polymer chain aligns along the *c*‐axis, the *d*‐spacing distance of the (001) planes (0.71 nm) represent the length of the polymer repeat unit. Based on the dimension of 29.7 nm along the [001] direction (Table S2), the average number of repeat units in Li_2_DHBQ was estimated to be 42, corresponding to a number average molecular weight of 7900 Daltons. SEM images (Figure 1c) show that the as‐prepared Li_2_DHBQ is predominantly composed of large rod‐like particles, measuring approximately 2–5 μm in length and 0.5–3 μm in width.

Li_2_DHBQ is soluble in DI water. The UV‐Vis spectrum of the Li_2_DHBQ aqueous solution reveals a prominent absorption peak at 315 nm (Figure 1d). To assess its solubility in the electrolyte, 1 M LiTFSI in DOL/DME (1 : 1), an excess amount of Li_2_DHBQ was added and stirred for 24 h at room temperature. The UV‐Vis spectrum of this sample shows extremely weak absorption (absorbance <0.01) attributable to Li_2_DHBQ, indicating its very low solubility in this electrolyte (<5.8×10^−5^ g L^−1^), based on the molar extinction coefficient of Li_2_DHBQ of 2.9×10^4^ L^−1^ mol^−1^ cm^−1^ at 315 nm.^[18]^

### 3.2 Electrochemical Properties

#### 3.2.1 Determination of Voltage Ranges for Redox Reactions of Li_2_DHBQ and SEI Layer Formation

**Figure 2:**
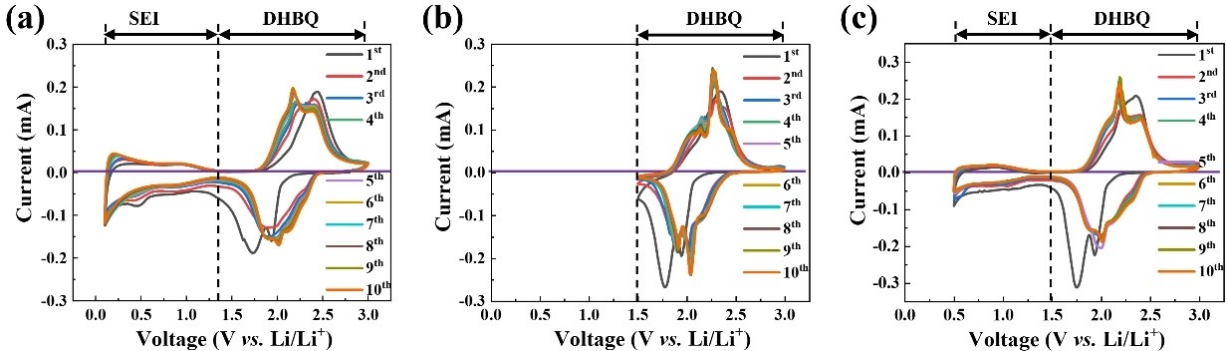
CV diagrams of a Li_2_DHBQ electrode at a scan rate of 0.1 mV s^−1^ in different voltage window ranges: (a) 0.1–3.0 V, (b) 1.5–3.0 V, and (c) 0.5–3.0 V.

Cyclic voltammetry (CV) was employed to investigate the voltage ranges for redox reactions and the SEI layer formation in the Li_2_DHBQ electrode. The redox reactions of Li_2_DHBQ are expected to occur within the voltage window of approximately 1.5–3.0 V with respect to the Li/Li^+^ electrode,^[19]^ whereas the formation of the SEI layer takes place within the voltage range of approximately 0–1.5 V.[^38^, ^39^] Therefore, a coin cell consisting of a Li_2_DHBQ cathode (60 % Li_2_DHBQ, 30 % SP, 10 % SA), a lithium foil anode, and a 1 M LiTFSI electrolyte in DOL/DME was initially scanned across a voltage range of 0.1–3.0 V to determine voltage ranges of both the redox reactions of Li_2_DHBQ and the formation of the SEI layer on the cathode. As shown in Figure 2a, during the first reduction scan, a peak at 1.96 V appeared, followed by a strong peak at 1.76 V, which correspond to the reduction reactions of the quinone groups in the DHBQ anions.^[19]^ After the completion of the DHBQ reduction (at around 1.25 V), the cathodic current gradually increases with decreasing scanning voltage until ca. 0.6 V, indicating the formation of the SEI layer involving the decomposition of the solvents (DOL and DME), which occurs at potentials below ca. 1.5 V.[^38^, ^39^] Subsequently, the cathodic current increases rapidly and reaches a peak value at 0.42 V, which corresponds to the decomposition of LiTFSI.^[40]^

In the subsequent oxidation scan, the capacity within the voltage range of 0.1–1.25 V is partially recovered, which is considered the capacitive effect of the electrode in this low voltage range.^[41]^ Oxidation reactions of the reduced DHBQ species start to occur at ca. 1.8 V, reaching an anodic current peak at 2.45 V, and are almost completed at 2.8 V. The second cathodic scan shows a broad peak centered at 1.95 V, with the overall profile shifted to higher potentials compared to the first cathodic scan. The second anodic profile for the reduced DHBQ species shifts to lower potentials with two peaks at 2.20 and 2.41 V, respectively, indicating a reduction in the diffusion barrier for Li^+^ ions. This shift may be attributed to the cracking of Li_2_DHBQ particles during the first scan, as revealed by the subsequent SEM analysis. SEI formation continues within the potential range of 0.1–1.25 V, albeit to a lesser extent. After the second scan, the redox profile for the DHBQ species stabilizes, while SEI formation (represented by the decomposition of LiTFSI at ca. 0.4 V) is completed after about five cycles.

Based on the CV survey scans discussed above, we narrowed the potential window to the range of 1.5–3.0 V to focus on the reduction reactions of DHBQ species without interference from SEI formation. As shown in Figure 2b, two distinct reduction peaks at 1.94 V and 1.77 V are observed, corresponding to the reduction of the DHBQ ligand during the initial cycle. The oxidation peak for the reduced DHBQ species appears at 2.36 V during the subsequent anodic scan. After several cycles, the shape of the curve remains largely unchanged, though the reduction and oxidation peaks draw closer due to the activation process. This indicates that the reversible redox reactions of DHBQ occur between 1.5 V and 3.0 V. Overall, the DHBQ reduction reactions occur similarly in this voltage window as in the 0.1–3.0 V range.

Next, we scanned another fresh Li_2_DHBQ electrode in the voltage range of 0.5–3.0 V to reduce the thickness and the inorganic content of the SEI layer on the cathode compared to the one cycled in the 0.1–3.0 V range. As shown in Figure 2c, the DHBQ redox profiles remain similar to those obtained in the broader (0.1–3.0 V) and narrower (1.5–3.0 V) voltage ranges, while the areas of the CV curve region representing the SEI formation with respect to the areas of the DHBQ redox reactions are smaller than those of the curves scanned in the 0.1–3.0 V range. Notably, the peak representing the decomposition of LiTFSI is only partially revealed, which may result in a more organic‐rich SEI layer. This partial peak almost disappeared after the third scan, whereas it disappeared after the fifth scan for the electrode scanned in the 0.1–3.0 V range. The earlier termination of LiTFSI decomposition, along with the smaller CV areas in the SEI formation range, indicates that the SEI layer formed within the 0.5–3.0 V range would be thinner than that formed in the 0.1–3.0 V range.

#### 3.2.2 Cell Performance of Li_2_DHBQ Electrodes Cycled Across Various Voltage Windows

The galvanostatic discharge/charge performances of Li_2_DHBQ electrodes were assessed across different voltage windows, maintaining the same charge cutoff voltage of 3.0 V while varying the discharge cutoff voltages of 1.5, 1.0, 0.7, 0.5, and 0.1 V, respectively. These varied cutoff voltages are expected to form SEI layers with differing thicknesses and compositions, potentially influencing the battery′s capacity and cycling stability. Given that the redox reactions of the DHBQ species predominantly occur between 1.5 V and 3.0 V, our analysis focuses on specific capacity values within this range to ensure a fair comparison of batteries with different discharge cutoff voltages.

**Figure 3:**
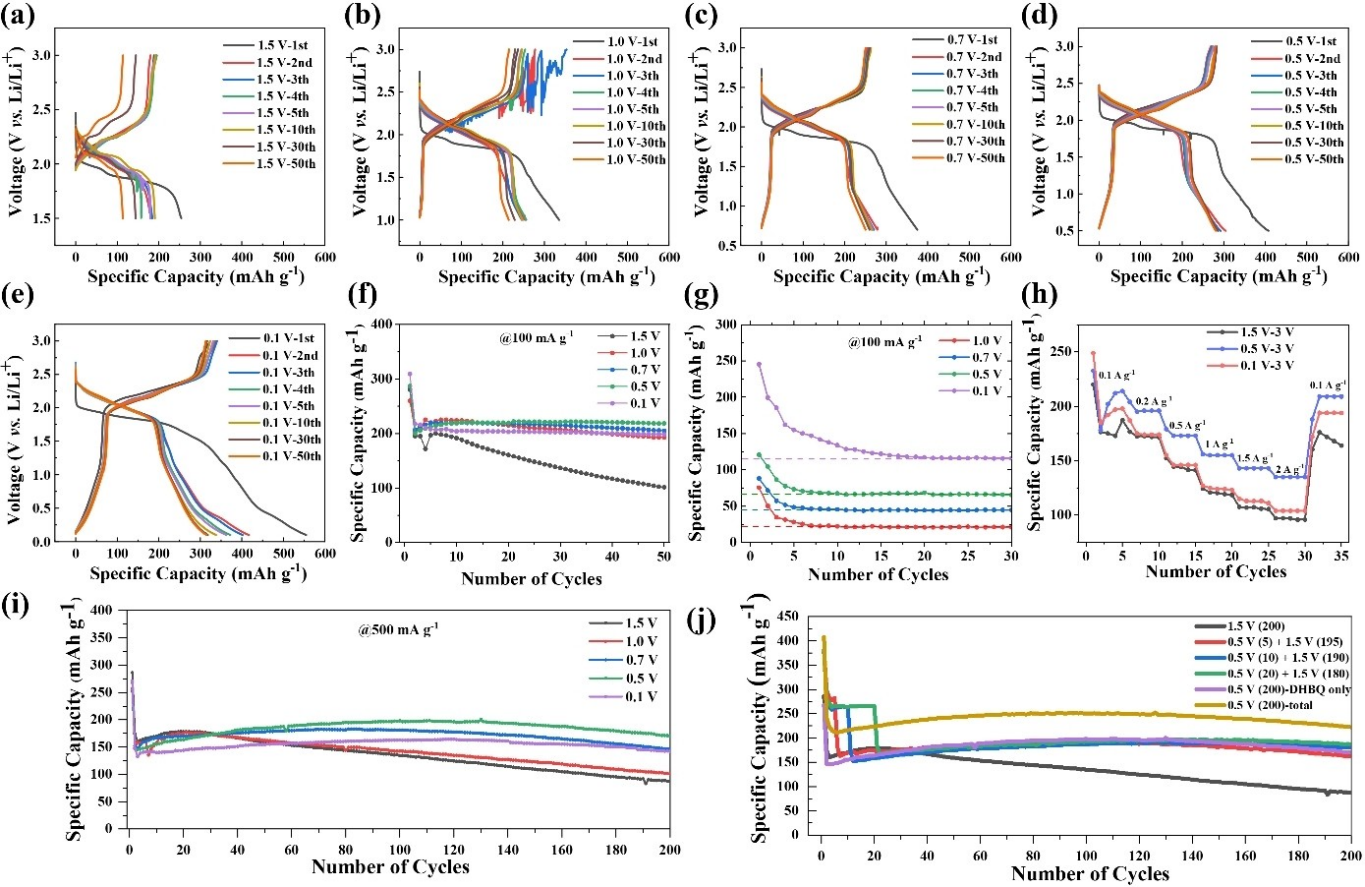
(a)–(e) Galvanostatic discharge/charge performance of Li2DHBQ cathodes with discharge cutoff voltages of 1.5, 1.0, 0.7, 0.5, and 0.1 V at a rate of 100 mA g^−1^ at different cycles; (f) Cycling performance of the batteries with different cutoff discharge voltages at a rate of 100 mA g^−1^ for 50 cycles, where the discharge capacity values from 3.0 V–1.5 V, corresponding to the reduction of DHBQ ligands, are used; (g) Discharge capacities of the batteries from 1.5 V to different discharge cutoff voltages at a rate of 100 mA g^−1^ for 30 cycles, attributed to SEI formation and the capacitive effect; (h) C‐rate performance of batteries with different discharge cutoff voltages; (i) Long‐term stability at a rate of 500 mA g^−1^, where the discharge capacity values from 3.0 V–1.5 V, corresponding to the reduction of DHBQ ligands, are used (the initial cycle was conducted at 100 mA g^−1^ for activation); (j) Long‐term stability of batteries at 500 mA g^−1^ from 1.5 V–3.0 V with different numbers of initial cycles at a rate of 100 mA g^−1^ from 0.5 V–3.0 V for SEI formation, along with batteries cycled in the voltage ranges of 0.5–3.0 V (0.5 V (200)) and 1.5–3.0 V (1.5 V (200)) at a rate of 500 mA g^−1^, where for the 1.5 V (200) battery, both the total and contributions from DHBQ only are plotted.

Figures 3a–e show the selected discharge/charge curves of the batteries cycled at a current density of 100 mA g^−1^ and Figure 3f displays their cycling performance over 50 cycles. These batteries exhibit high initial discharge capacities over 260 mAh g^−1^ within the range of 3.0 V–1.5 V, corresponding to the reduction of DHBQ species. All batteries experienced a significant drop in capacity in the second cycle, decreasing to 195–218 mAh g^−1^, along with a notable increase in the discharge voltage. This is most likely due to the formation of cracks in the Li_2_DHBQ particles during the first cycle, as mentioned earlier. The battery cycled within the voltage range of 1.5–3.0 V shows the fastest capacity decay, retaining only 102 mAh g^−1^ (36 % of the initial capacity) after 50 cycles. Lowering the discharge cutoff voltage to 1.0 V and 0.7 V results in greater stability, with capacities of 193 mAh g^−1^ (68 % retention) and 206 mAh g^−1^ (72 % retention), respectively, after 50 cycles. At a discharge cutoff voltage of 0.5 V, the battery demonstrates even higher stability, maintaining 218 mAh g^−1^ (76 % retention) after 50 cycles. However, with a discharge cutoff voltage of 0.1 V, the capacity drops to 198 mAh g^−1^ (69 % retention) after 50 cycles. Clearly, the battery cycled within the voltage window of 0.5–3.0 V exhibits the best cycling stability.

Due to significant structural changes or crack formation in the Li_2_DHBQ particles during the first cycle, resulting in a drastic capacity drop in the second cycle and subsequent capacity fluctuations, it is more appropriate to assess the cycling stability of these batteries from the 6^th^ cycle onwards. Between the 6^th^ and the 50^th^ cycles, the capacity retention and decay rates are calculated to be 50.8 %, 86.5 %, 93.6 %, 101.8 %, and 95.8 % for batteries with discharge cutoff voltages of 1.5, 1.0, 0.7, 0.5, and 0.1 V, respectively. Remarkably, the battery with a 0.5 V discharge cutoff voltage showed almost no change in capacity from the 6^th^ to the 50^th^ cycle.

Figure 3g shows the discharge capacities below 1.5 V for batteries with discharge cutoff voltages of 1.0, 0.7, 0.5, and 0.1 V at 100 mA g^−1^ within the first 30 cycles, which are contributed by SEI formation and the capacitive effect of SP. The capacities stabilize at 22, 45, 63, and 116 mAh g^−1^ after the 7^th^, 8^th^, 9^th^, and 21^st^ cycles, respectively, corresponding to their capacitive capacities. The excess capacities before stabilization are attributed to SEI formation in the early cycles.

The rate performances of batteries with discharge cutoff voltages of 1.5, 0.5, and 0.1 V were assessed using various current densities, ranging from 0.1 A g^−1^–2 A g^−1^, as depicted in Figure 3h. Notably, the battery with the 0.5 V discharge cutoff voltage exhibits the best rate performance. It maintains a capacity of 214 mAh g^−1^ at a current density of 0.1 A g^−1^, sustains 135 mAh g^−1^ at 2 A g^−1^ (approximately 7 C), and rebounds to 209 mAh g^−1^ at 0.1 A g^−1^ between the 30^th^ and 35^th^ cycles. In contrast, batteries with discharge cutoff voltages of 1.5 V and 0.1 V display notably inferior rate performance. While the battery with the 0.1 V discharge cutoff voltage recovers and remains stable when cycled again at 0.1 A g^−1^, the one with the 1.5 V discharge cutoff voltage experiences a rapid capacity decline when the current density is reset to 0.1 A g^−1^.

The long‐term stability of the batteries was evaluated by cycling them at 500 mA g^−1^ with different discharge cutoff voltages (refer to Figure S4a–e for their discharge/charge curves). As depicted in Figure 2i, the battery with a 1.5 V discharge cutoff voltage experiences an increase in capacity after the second cycle until the 19^th^ cycle before declining rapidly. After 200 cycles, its capacity is 87 mAh g^−1^, corresponding to a retention rate of 53 % relative to the capacity at the 6^th^ cycle, with a capacity decay rate of 0.28 % per cycle between the 19^th^ and 200^th^ cycles.

As the discharge cutoff voltage decreases, the early capacity rising stage progressively extends to the 21^st^, 81^st^, 111^th^, and 120^th^ cycle for batteries with discharge cutoff voltages of 1.0, 0.7, 0.5, and 0.1 V, respectively. Their capacities at the 200^th^ cycle are 101, 146, 170, and 142 mAh g^−1^, corresponding to retention rates of 63 %, 91 %, 115 %, and 101 %, respectively, markedly improved compared to the battery with a discharge cutoff voltage of 1.5 V. The capacity decay rates calculated after they reached peak values are 0.24 % (with the 1.0 V discharge cutoff voltage), 0.18 % (with the 0.7 V discharge cutoff voltage), 0.16 % (with the 0.5 V discharge cutoff voltage), and 0.17 % per cycle (with the 0.1 V discharge cutoff voltage). Once again, the battery with the 0.5 V cutoff voltage demonstrates the most robust cycling performance.

According to Figure 2g, SEI formation reactions primarily occur within the initial 20 cycles at 100 mA g^−1^ for batteries with a discharge cutoff voltage below 1.0 V. Therefore, it is plausible that a robust SEI layer can be formed to protect the Li_2_DHBQ morphology by cycling the battery for a few cycles at a lower discharge cutoff voltage before switching to a normal discharge cutoff voltage of 1.5 V. Accordingly, we cycled batteries in the voltage range of 0.5–3.0 V for 5, 10, and 20 cycles at 100 mA g^−1^, followed by cycling in the range of 1.5–3.0 V at 500 mA g^−1^. As shown in Figure 2j, all three batteries exhibit significantly improved stability, with capacities of 163, 181, and 187 mAh g^−1^ at the 200^th^ cycle, respectively, compared to the battery cycled only in the range of 1.5–3.0 V, which retained a capacity of 87 mAh g^−1^. Notably, the batteries cycled in the range of 0.5–3.0 V for the initial 10 and 20 cycles demonstrate even better long‐term stability than the one cycled continuously in the range of 0.5–3.0 V, which exhibits a capacity of 170 mAh g^−1^ at the 200^th^ cycle.

### 3.3 SEI Layer Formation and its Impact on Battery Performance

#### 3.3.1 Relationship between Morphological Changes of Li_2_DHBQ Electrodes and Discharge Cutoff Voltage

**Figure 4:**
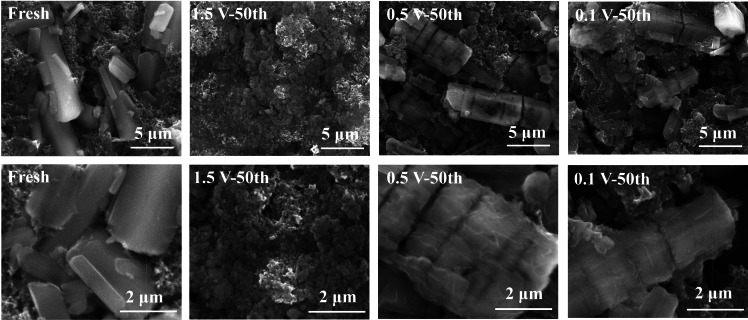
SEM images of a fresh Li_2_DHBQ electrode and those obtained after 50 cycles (in the charged state) at a current density of 100 mA g^−1^ with discharge cutoff voltages of 1.5, 0.5, and 0.1 V, respectively.

Our previous research on another DHBQ compound, Cu‐DHBQ, has demonstrated that morphological damage or pulverization of Cu‐DHBQ can significantly undermine battery stability.^[18]^ As shown in the previous section, the Li_2_DHBQ battery utilizing 10 % SA as the binder failed to achieve battery stability when cycled within the voltage window of 1.5–3.0 V that allows the redox reactions of Li_2_DHBQ only. We suspect that the rapid degradation of this battery is also linked to morphological damage to the active material Li_2_DHBQ. This assertion is supported by SEM images shown in Figure 4 and Figure S6, depicting the Li_2_DHBQ electrode cycled between 1.5–3.0 V for 50 cycles at 100 mA g^−1^. These images reveal significant pulverization of the active materials, with no discernible micron‐sized Li_2_DHBQ particles that were present in the fresh electrode. This pulverization leads to a notable disconnection of the active material from the conductive carbon, consequently accelerating capacity degradation.

In contrast, the electrode cycled within the range of 0.5–3.0 V exhibits minimal alteration in the size and shape of the active materials after 50 cycles, albeit with some fractures along the rods’ cross‐sectional direction. Furthermore, compared to the smooth surface of Li_2_DHBQ particles in the fresh electrode, those in the cycled electrode feature a roughened surface with evident thin amorphous coatings, indicative of the SEI layer. The morphology of the Li_2_DHBQ particles in the electrode cycled within the range of 0.1–3.0 V for 50 cycles is also well maintained, although they display depressions on the Li_2_DHBQ surface, altering its shape from rod‐like to bamboo‐like structure. Furthermore, the fractures on the Li_2_DHBQ particles are less pronounced, possibly due to the presence of a thicker SEI layer, compared to those observed in the electrode cycled within the range of 0.5–3.0 V. These results demonstrate the significant impact of discharge cutoff voltage on the morphological stability of Li_2_DHBQ and on battery cycling stability.

**Figure 5:**
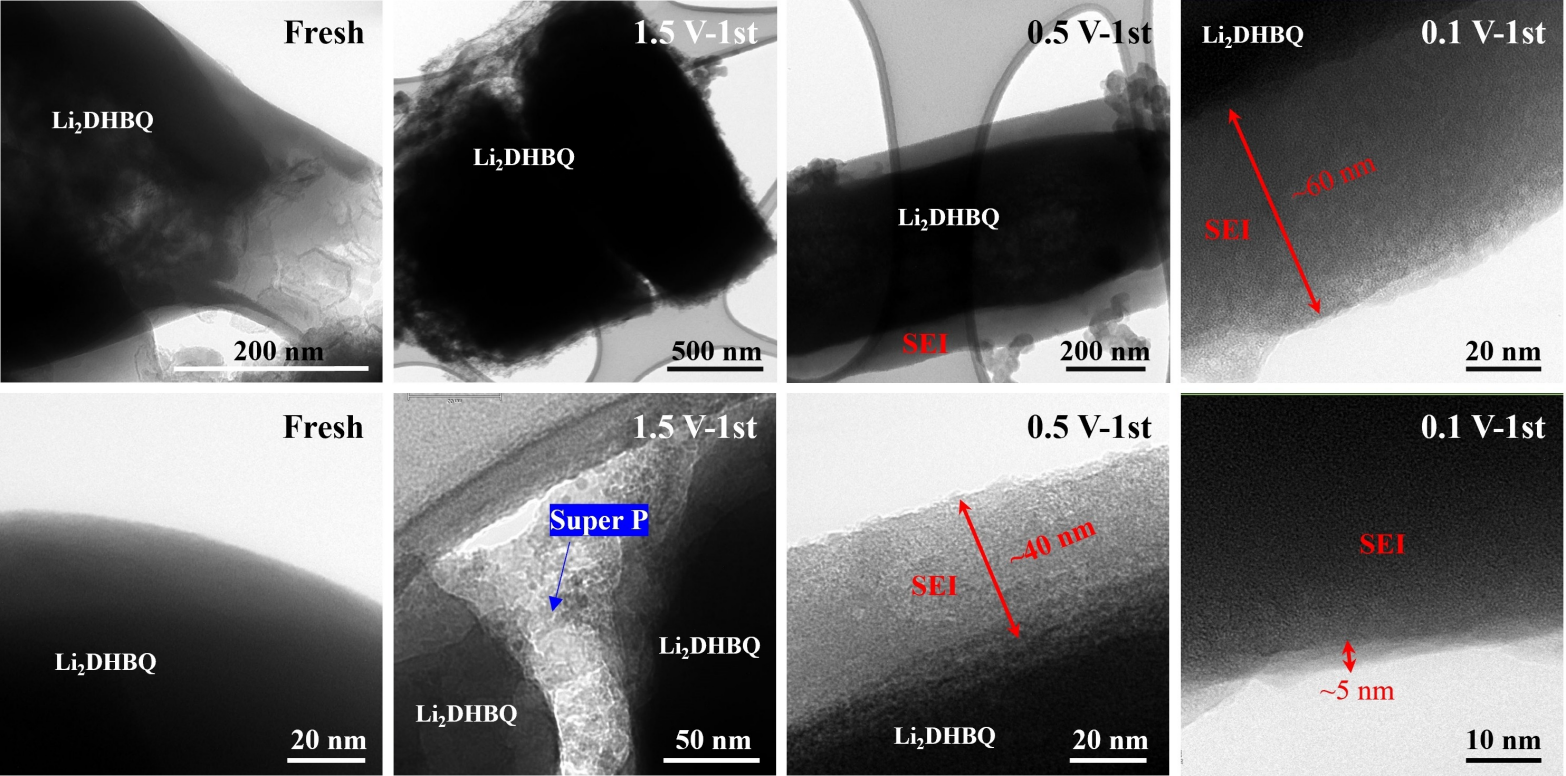
TEM images of a fresh Li_2_DHBQ electrode and those obtained after the first cycle (in the discharged state) at a current density of 0.1 A g^−1^ with discharge cutoff voltages of 1.5 V, 0.5 V, and 0.1 V, respectively.

To confirm the formation of an SEI layer on the surface of the active material particles, we conducted measurements on the electrodes after the initial discharge using a transmission electron microscope (TEM) (refer to Figure 5). In both the fresh electrode and the electrode with a 1.5 V discharge cutoff voltage, the Li_2_DHBQ particles exhibit smooth surfaces without any discernible SEI layer. Conversely, the electrode subjected to a 0.5 V discharge cutoff voltage exhibits a light‐colored smooth layer with a thickness of ca. 40 nm on the surface of the active materials, indicative of the SEI layer. In the case of the electrode with a 0.1 V discharge cutoff voltage, the SEI layer is noticeably thicker at ca. 60 nm. Additionally, an uneven thin layer measuring ca. 5 nm in thickness emerges on the surface of the SEI layer, which may reflect its more brittle inorganic‐rich composition (refer to the subsequent XPS data).

We performed electrochemical impedance spectroscopy (EIS) and measurements on a Li_2_DHBQ battery at different charged and discharged states during the first cycle (Figure S5 and Table S3). The Nyquist plots in Figure S5 reveal that the fresh battery displays a semicircle in the high‐to‐medium frequency region, where the real part of the impedance corresponds to the charge transfer resistance (R_ct_). In the low‐frequency region, the plot shows a straight‐line slope greater than 45°, resembling a finite space Warburg (FSW) element model, indicating a significant lithium‐ion diffusion barrier due to the densely packed pristine active material.^[42]^

The high‐to‐medium frequency response varies with the discharge/charge voltage, reflecting the resistance associated with solid‐electrolyte interphase (SEI) layer formation (R_SEI_). Since the semicircles corresponding to R_SEI_ and R_ct_ often overlap, separating their individual contributions is challenging. Thus, we calculated the total resistance, incorporating both R_ct_ and R_SEI_, using the appropriate fitting models (Table S3) and plotted it against the discharge/charge voltage (Figure S5c).

During discharge from open‐circuit voltage (OCV) to 1.5 V and 1.0 V, the total resistance decreases from 135.5 Ω to 116.3 Ω and further to 98.0 Ω. As minimal SEI formation is expected above 1 V, this decrease is attributed to the lithiation of Li_2_DHBQ, which induces structural damage, enhances charge transfer, and reduces R_ct_. However, as the battery is further discharged to 0.5 V and 0.1 V, the resistance increases to 110.2 Ω and 155.4 Ω, respectively. Since R_ct_ alone is not expected to rise at these low voltages, the increase is attributed to progressive SEI layer formation during deeper discharge. Upon recharging from 0.1 V–3.0 V, the resistance slightly decreases to 141.8 Ω, likely due to additional structural damage caused by volume contraction.

The low‐frequency region also exhibits changes with charge/discharge states, reflecting lithium‐ion diffusion behavior. At 1.0 V and 0.5 V, the impedance lines resemble a semi‐infinite length Warburg (SIW) model, characterized by a slope of 45°. However, at 0.1 V and after recharging to 3.0 V, the impedance plots bend towards the real axis, resembling a finite length Warburg (FLW) model.^[42]^ This shift indicates a transmissive electrode/electrolyte interface, suggesting enhanced lithium‐ion intercalation/deintercalation due to crystal damage in the Li_2_DHBQ material.

These EIS findings align well with the observed electrochemical performance, as well as the XRD (discussed below) and TEM results.

#### 3.3.2 Analysis of SEI Layer Composition by XPS

**Figure 6:**
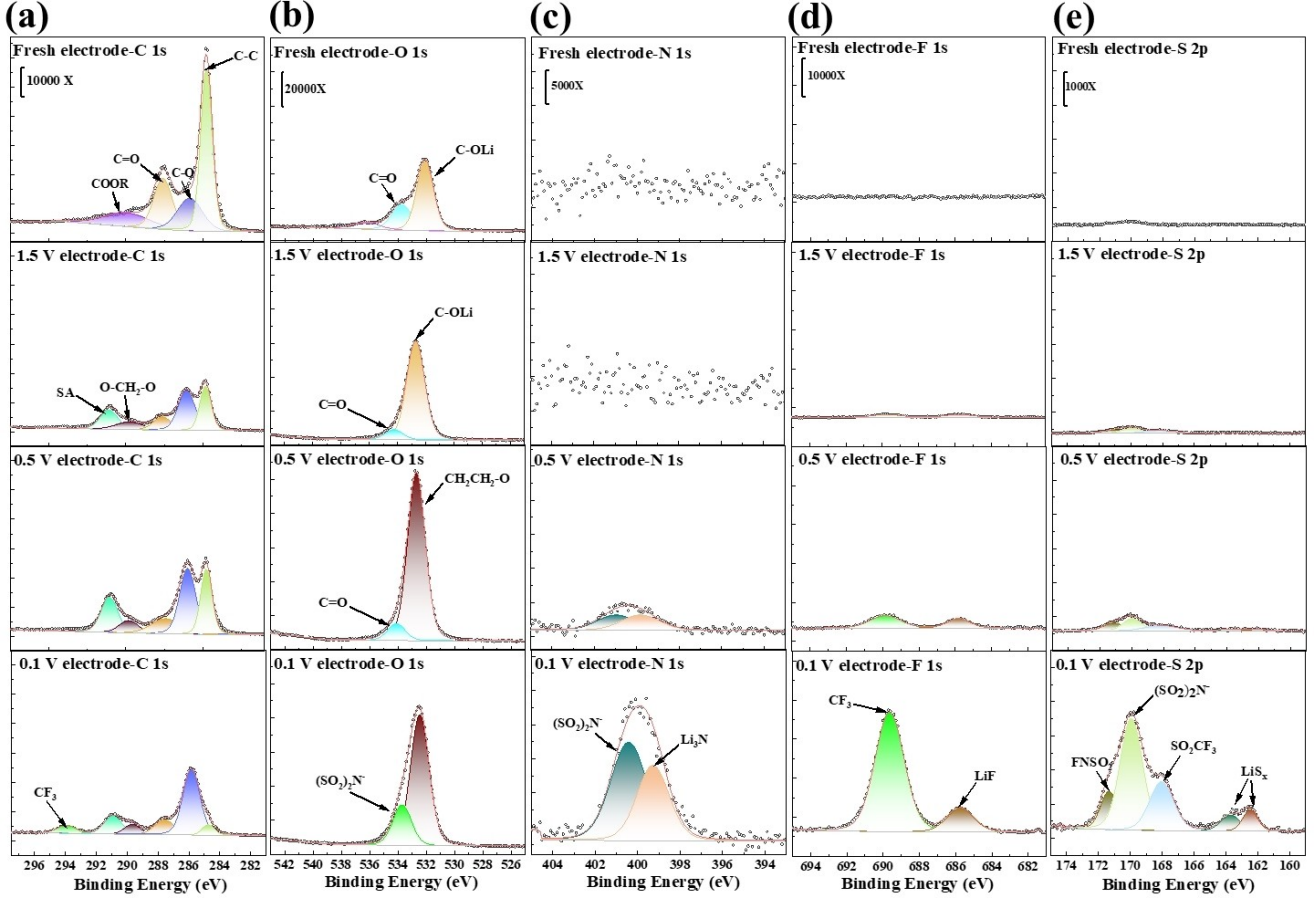
XPS results of fresh and 1^st^ discharging of Li_2_DHBQ cathodes under a current density of 100 mA g^−1^ with 1.5 V/0.5 V/0.1 V discharge cutoff voltage: (a) C 1s, (b) O 1s, (c) N 1s, (d) F 1s, (e) S 2p.

The SEI layer compositions of four Li_2_DHBQ electrodes, a fresh one and those discharged to 1.5 V, 0.5 V, and 0.1 V, respectively, were analyzed using XPS. Key peak assignments are shown in Table S4, and the SEI formation reactions are provided in Scheme S1. As shown in Figure 6a, the C 1s spectrum of the fresh cathode shows four main peaks corresponding to C(−O)OR of SA (289.9 eV),[^43^, ^44^] C−O and C−O of DHBQ and SA (287.6 and 285.8 eV),[^45^, ^46^, ^47^] and C−C (284.8 eV) in all components including SP in the electrode.^[46]^ Upon discharging to 0.5 V, two new peaks emerge at 289.8 eV and 291.0 eV, respectively. The former originates from the O−CH_2_−O from the polymeric product RO(CH_2_CH_2_OCH_2_O)_n_Li formed from DOL decomposition,^[48]^ while the latter is from the decomposition product of DME, RCO_2_Li.[^44^, ^49^] Additionally, the relative contents of C−C and C−O decrease, while that of C−O increases. These changes are likely due to the lithiation of DHBQ and the formation of a C−O rich SEI layer resulting from the decomposition of DOL and DME.[^38^, ^48^] After discharging to 0.1 V, the electrode exhibits a new peak at 294.0 eV, corresponding to −CF_3_, indicative of LiTFSI decomposition.[^38^, ^39^, ^49^, ^50^, ^51^] These XPS C 1s spectra suggest that an SEI layer is primarily composed of organic products formed via decomposition of DOL and DME between 0.5 V and 1.5 V, while it contains additional components produced via decomposition of LiTFSI between 0.1 V and 3 V. The peaks of the electrode discharge to 1.5 V are similar to the one discharged to 0.5 V, but with lower intensities. This indicates that the solvent decomposition mainly occurs below 1.5 V.

The O 1s spectrum of the fresh cathode shows two main peaks (534.2 and 532.7 eV) corresponding to C−O and C−OLi of Li_2_DHBQ, respectively.[^52^, ^53^] After discharging to 1.5 V, the peak intensity of C−OLi increases, likely due to lithiation of the quinone groups in DHBQ. Upon discharging the electrode to 0.5 V, there is a significant increase in the intensity of the peak at 532.7 eV, attributed to DOL decomposition and the formation of an O‐rich SEI layer (−CH_2_−CH_2_−O−).^[48]^ Once discharged to 0.1 V, the peak at 534.2 eV becomes much stronger, indicating the decomposition of LiTFSI to form (SO_2_)N^−^.^[54]^

Notably, the formation of (SO_2_)N^−^ only occurs below 0.5 V. Conversely, the relative content of O‐rich SEI layer (−CH_2_−CH_2_−O−) increases as the discharge cutoff voltage decreases from 1.5 V–0.5 V and further to 0.1 V. The results reaffirm that electrolyte solvents begin to decompose at voltages above 0.5 V, followed by the decomposition of LiTFSI at lower voltages below 0.5 V.

The N 1s spectra show no clear peaks for all cathodes except the one discharged to 0.1 V, which displays two peaks corresponding to (SO_2_)N^−^ (400.5 eV)^[54]^ and Li_3_N (399.5 eV),^[52]^ both originating from LiTFSI decomposition. Similar trends are observed in the F 1s and S 2p spectra. The F1s spectra of the electrode discharged to 0.1 V feature two main peaks corresponding to −CF_3_ (689.6 eV)^[49]^ and LiF (685.7 eV),^[55]^ arising from LiTFSI decomposition. In the S 2p spectra, a prominent peak at 170 eV ((SO_2_)N^−^) also appears for the electrode discharged to 0.1 V.^[56]^

Overall, these findings demonstrate that adjusting the discharge cutoff voltage between 1.5 V and 0.1 V enables the formation of an SEI layer with controllable thickness and composition on the surface of Li_2_DHBQ particles in the cathode, through electrolyte decomposition. The SEI layer formed at a discharge voltage of 0.5 V provides optimal thickness and composition, enhancing capacity and cycling stability by preserving the morphology of the active material.

#### 3.3.3 Study of Structural Changes of Li_2_DHBQ by XRD

**Figure 7:**
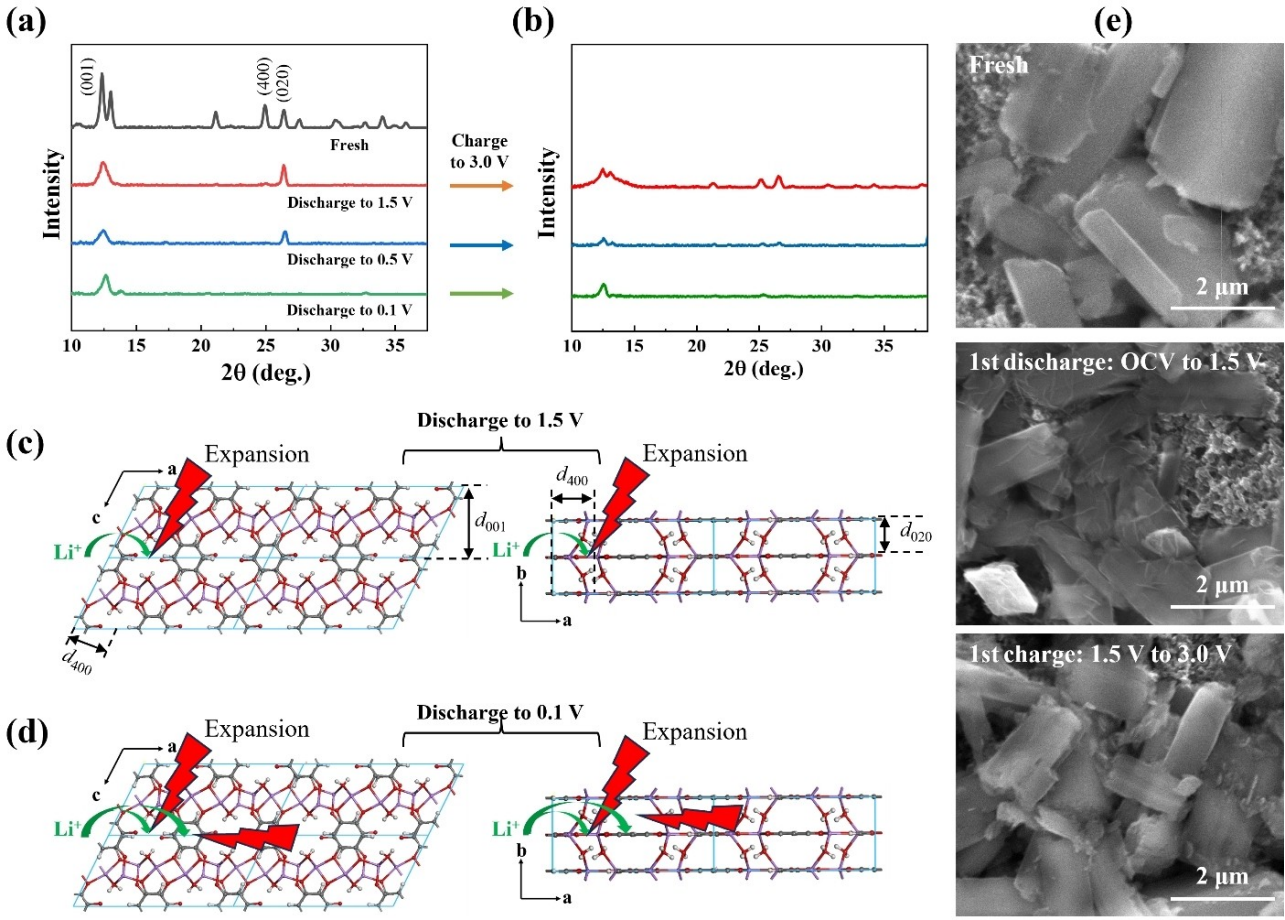
(a) XRD patterns of Li_2_DHBQ cathodes in fresh and discharged states at various voltages; (b) XRD patterns of Li_2_DHBQ cathodes recharged from different discharged states to 3 V; (c) Schematic illustration of lithiation at quinone sites within the Li_2_DHBQ crystal structure during discharge from the open‐circuit voltage (OCV) to 1.5 V, resulting in expansion along the *a*‐axis and disappearance of the (400) peak; (d) Schematic illustration of lithiation at quinone sites (mainly from OCV to ~1.5 V) and intercalation into benzene rings (primarily from ~0.5–0.1 V) within the Li_2_DHBQ crystal structure during discharge to 0.1 V, leading to expansion along both *a*‐ and *c*‐axes and disappearance of the (400) and (020) peaks, respectively; (e) SEM images of Li_2_DHBQ cathodes in the discharged and (re)charged states: fresh (in the charged state), discharged from the open circuit voltage (OCV) to 1.5 V, (re)charged from 1.5 V–3.0 V in the first cycle under a current density of 100 mA g^−1^ within the 1.5– 3.0 V voltage range.

To investigate the impact of discharge/charge processes on the crystal structure Li_2_DHBQ, we performed XRD measurements on batteries with different cut‐off voltages in their first cycle. As shown in Figure 7, the disappearance of the (400) peak after discharging to 1.5 V suggests disordering caused by lithiation at the quinone group, leading to expansion along the a‐axis (Figure 7c). When discharged to 0.1 V, the (020) peak also disappears, likely due to lithium intercalation on the benzene rings,^[17]^ causing expansion along the c‐axis (π‐π stacking direction) (Figure 7d). Even at 0.5 V, some degree of lithium intercalation would occur.^[17]^

Meanwhile, the (001) peak remains strong across all samples, indicating that the polymer chains remain relatively stable since no lithiation occurs along the b‐axis. Upon recharging to 3 V, partial recovery of the (400) peak is observed for the cathode discharged to 1.5 V. However, the (400) peaks for cathodes discharged to 0.5 V and 0.1 V do not recover, and other peaks are also weakened compared to their discharged states. This suggests that delithiation causes contraction along the (400) and (020) directions (for cathodes discharged to 0.1 V and 0.5 V), leading to further crystal structure damage.

These crystal structure damages of Li_2_DHBQ are closely linked to its morphological changes caused by cycling. As shown in Figure 7e, the cathode discharged to 1.5 V in the first cycle exhibits exfoliated layers along the longitudinal direction of the Li_2_DHBQ rods, markedly different from the smooth, rod‐like morphology of the fresh sample. As discussed earlier, the reduction of quinone groups induces expansion along the a‐axis, which leads to particle exfoliation. Upon recharging to 3 V, the layered structure disappears due to delithiation and partial restoration of the crystal structure. However, the surfaces of the Li_2_DHBQ particles become noticeably rougher compared to the fresh sample, indicating permanent damage to the crystal structure. These XRD results indicate that the significant chnages in the CV profiles and the drop in capacity during the second cycle are primarily caused by damage to the crystal structure during the first cycle in cells with different cutoff voltages.

After extensive cycling for 50 cycles, the cathode discharged to 1.5 V becomes pulverized due to repeated volume expansion and contraction (Figure 4). In contrast, for cells discharged to lower voltages (0.5 and 0.1 V), the formation of SEI layers on the Li_2_DHBQ particles helps confine the exfoliated layered structures during discharge. Although these particles experience more severe crystal structure damage, their overall morphologies remain well‐preserved. The dark cracks observed along the cross‐sectional direction of the rods in both the 0.5 V and 0.1 V discharged cathodes are likely due to the disruption of π‐π stacking interactions caused by intercalation, as indicated by the parallel alignment of the crack lines, consistent with the XRD data discussed above.

## 4 Conclusions

In conclusion, this study investigated the root cause for the rapid capacity decay of organic cathodes based on a quinone compound, Li_2_DHBQ. It was found that severe morphological damage to the active material, rather than the solubility, is primarily responsible for the poor battery cycling performance. By promoting the formation of an SEI layer on Li_2_DHBQ through reduction at lower discharge voltages, significant improvements in morphological stability were achieved, leading to enhanced battery performance. Specifically, a discharge cutoff voltage of 0.5 V resulted in an optimal SEI layer thickness and organic‐rich composition, enabling the battery to maintain a capacity of 170 mAh g^−1^ with a low decay rate of 0.16 % after 200 cycles within a voltage range of 0.5–3.0 V at 500 mA g^−1^. The efficacy of this approach was further demonstrated by a battery initially cycled with a discharge cutoff voltage of 0.5 V for 20 cycles to form an SEI layer, followed by cycling at a normal discharge cutoff voltage of 1.5 V at 500 mA g^−1^. This battery achieved even better cycling stability, maintaining a capacity of 187 mAh g^−1^ at the 200^th^ cycle. These are significant improvements compared to the battery cycled only in the normal range of 1.5–3.0 V, which retained a capacity of 87 mAh g^−1^. This innovative approach underscores the efficacy of SEI layer formation in bolstering the morphological resilience of organic cathode materials, thus facilitating their sustainable integration into future battery technologies.

## Conflict of Interests

The authors declare no conflict of interest.

5

## Supporting information

As a service to our authors and readers, this journal provides supporting information supplied by the authors. Such materials are peer reviewed and may be re‐organized for online delivery, but are not copy‐edited or typeset. Technical support issues arising from supporting information (other than missing files) should be addressed to the authors.

Supporting Information

## Data Availability

The data that support the findings of this study are available in the supplementary material of this article.
