# Supplementary material for: Effective Stabilization of Organic Cathodes Through Formation of a Protective Solid Electrolyte Interface Layer via Reduction
Source: ChemSusChem. 2024 Nov 25;18(7):e202401599. doi: 10.1002/cssc.202401599 (PMC11960584; doi:10.1002/cssc.202401599)
Supplement: Supplementary file 1 — Supporting Information [file CSSC-18-e202401599-s001.pdf]

ChemSusChem

Supporting Information

Effective Stabilization of Organic Cathodes Through Formation of a Protective Solid Electrolyte Interface Layer via Reduction

Yonglin Wang, Zhe Huang, Xiguang Gao, Razieh Fazaeli, and Yuning Li*

Supporting Information

Effective Stabilization of Organic Cathodes Through Formation of a Protective Solid Electrolyte Interface Layer via Reduction

Yonglin Wang, Zhe Huang, Xiguang Gao, Razieh Fazaeli and Yuning Li*

Department of Chemical Engineering and Waterloo Institute for Nanotechnology (WIN), University of Waterloo, Waterloo, 200 University Avenue West, Waterloo, Ontario N2L 3G1, Canada. Email: yuning.li@uwaterloo.ca

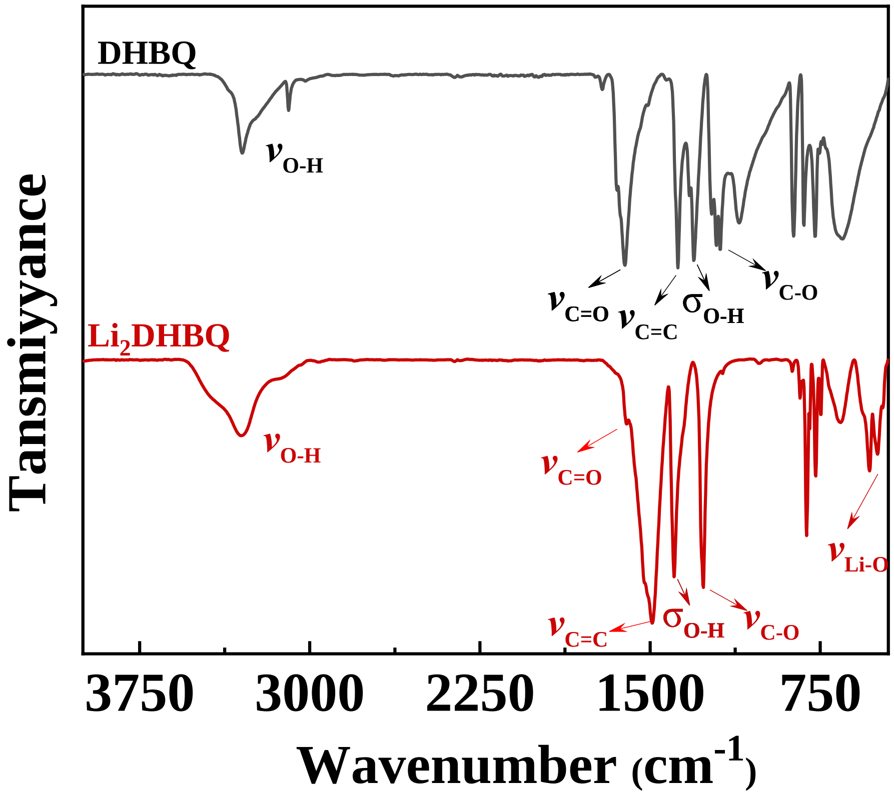

Figure S1. FTIR spectra of DHBQ and Li_2DHBQ.

Table S1. The assignment of the peaks in the FTIR spectra of DHBQ and Li_2DHBQ.

Vibration mode / cm^{-1}	DHBQ	Li_2DHBQ
O-H stretching	3298	3298
=C-H stretching	3093	3122
C=O stretching	1610	1604
C=C stretching	1377	1490
O-H bending (in-plane)	1307	1394
C-O stretching	1190	1267
C=C and =C-H bending	867, 821, 771, 648	839, 810, 769, 661
Li-O stretching	Absence	497

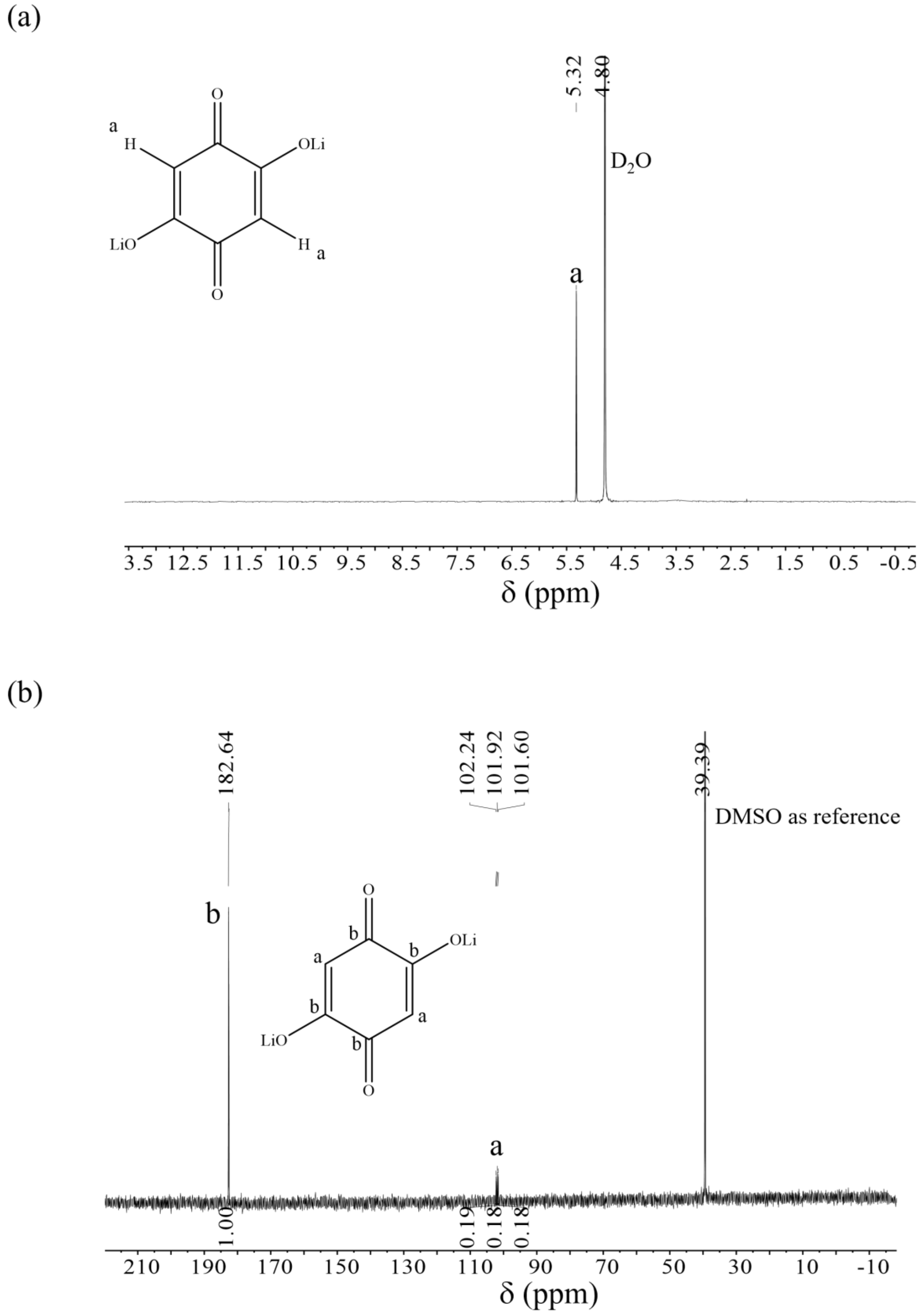

Figure S2. (a) 300 MHz ^{1}HNMR and (b) 75 MHz ^{13}CNMR spectra of Li_2DHBQ measured in D_2O. The chemical shifts were calibrated using residual DHO (4.80 ppm) for the ^{1}H NMR spectrum and a small amount of added dimethyl sulfoxide (DMSO) (39.39 ppm)[1] for the ^{13}C NMR spectrum.

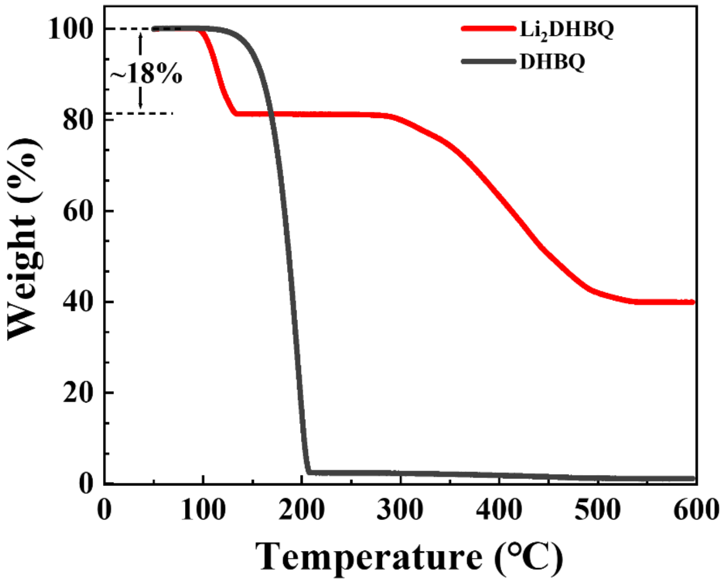

Figure S3. TGA curves of Li_2DHBQ and DHBQ.

Table S2. Single crystal parameters and grain size of Li_2DHBQ.

2θ (°)	d, nm	Planes	crystallite size, nm[a]
12.525	0.71	(001)	29.7
25.125	0.35	(400)	24.6
27.725	0.32	(020)	22.5

[a] Calculated using the Scherrer equation.

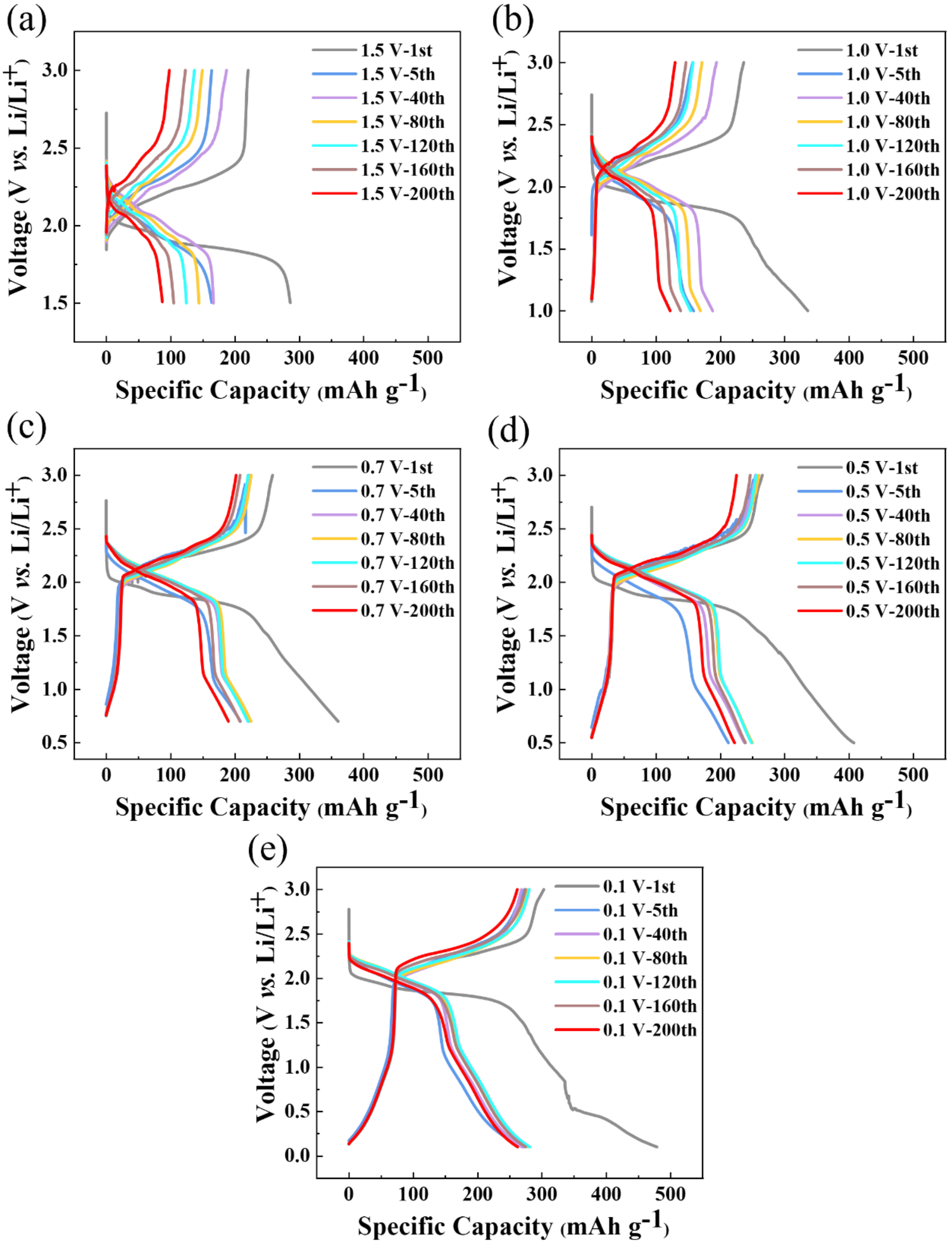

Figure S4. Galvanostatic discharge/charge performance of Li_2DHBQ cathodes under a current density of 500 mA g^{-1} with a charge cutoff voltage of 3.0 V and various discharge cut-off voltages (a) 1.5 V, (b) 1.0 V, (c) 0.7 V, (d) 0.5 V, and (e) 0.1 V at different cycles. The initial cycle was conducted at 100 mA g^{-1} for activation.

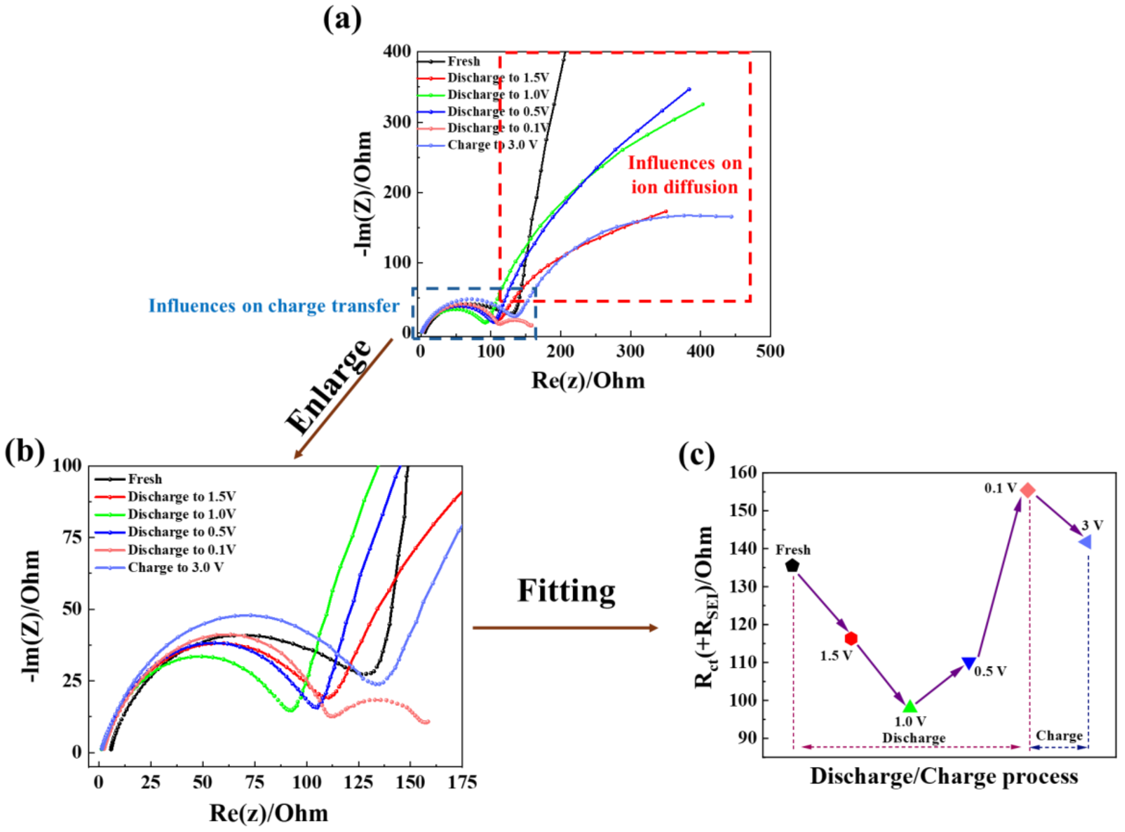

Figure S5. (a) EIS Nyquist plots of a Li_2DHBQ battery in different states during the first cycle, (b) enlarged charge transfer regions, and (c) the total resistance, comprising charge transfer resistance (R_{ct}) and SEI layer resistance (R_{SEI}), calculated from (b) using the fitting circuit models presented in Table S3.

Table S3. EIS fitting results of the charge transfer region of the Li_2DHBQ batteries obtained from EIS Nyquist plots in Figure S6.

States	R_s, Ω	$R_{ct}+R_{SEI}$, Ω	Fitting Model
Fresh	4.2	135.5	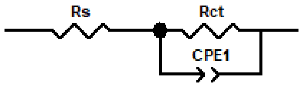
Discharge to 1.5 V	1.1	116.3	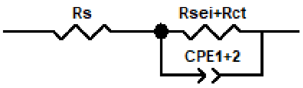
Discharge to 1.0 V	0.7	98.0	
Discharge to 0.5 V	0.9	110.2	
Discharge to 0.1 V	2.0	155.4 (114.1+41.3)	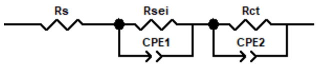
Cha to 3.0 V	0.9	141.8	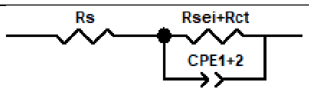

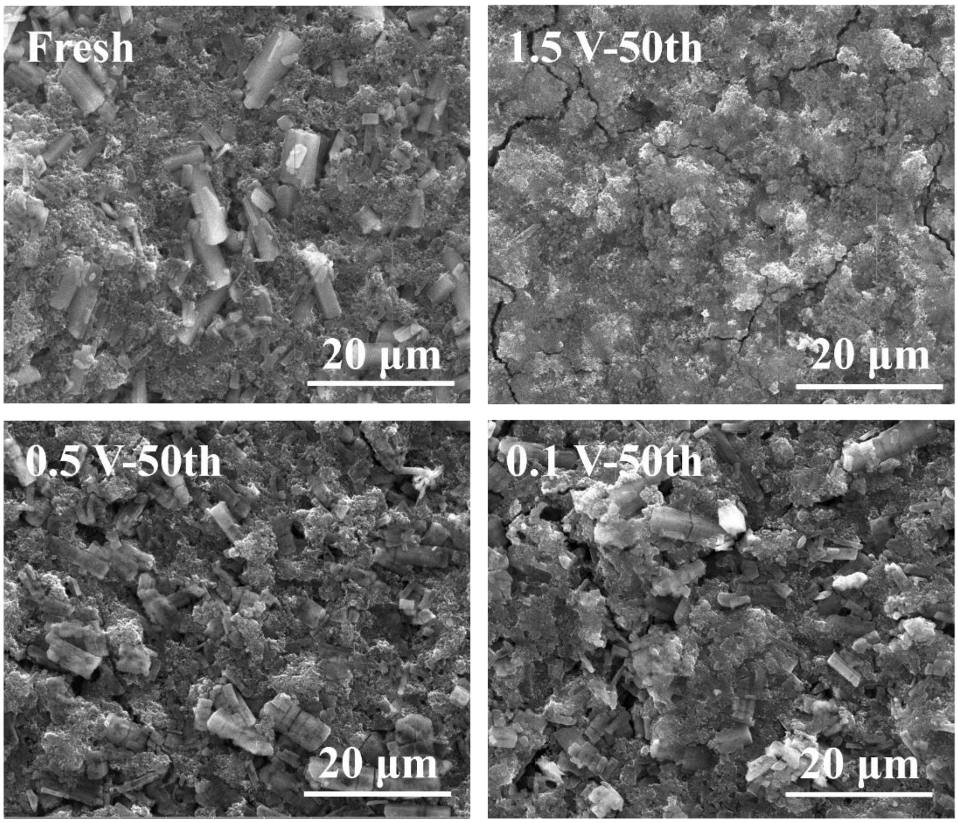

Figure S6. SEM images of fresh and 50th cycle of Li_2DHBQ cathodes under a current density of 100 mA g^{-1} with a charge cutoff voltage of 3.0 V and various discharge cut-off voltages 1.5, 0.5, and 0.1 V.

Table S4. XPS assignments of key components in the SEI layer formed from electrolyte decomposition (see **Scheme S1** for detailed reactions).

Reactant	Product	C 1s	O 1s	N 1s	F 1s	S 1s
DOL	$RO(CH_2CH_2OCH_2O)_nLi$	C-OR: 286[2] O-CH_2-O: 289.5[3]	C-OR: 532.2[2] O-CH_2-O: 531[3] CH_2-CH_2-O: 532.4[3]			
DME	RCO_2Li	290-291[4]				
LiTFSI	Li_3N			399.5[5]		
	Li_2S					Li2S: 161.5, 162.5[6]
	LiS_x					163.5 eV[7]
	LiF				685[8]	
	SO_2CF_3				689.9[9]	168[9]
	$(SO_2)_2N$-		534[10]	401[10]		170[10,11]
	$FNSO_2$					171.3[12]

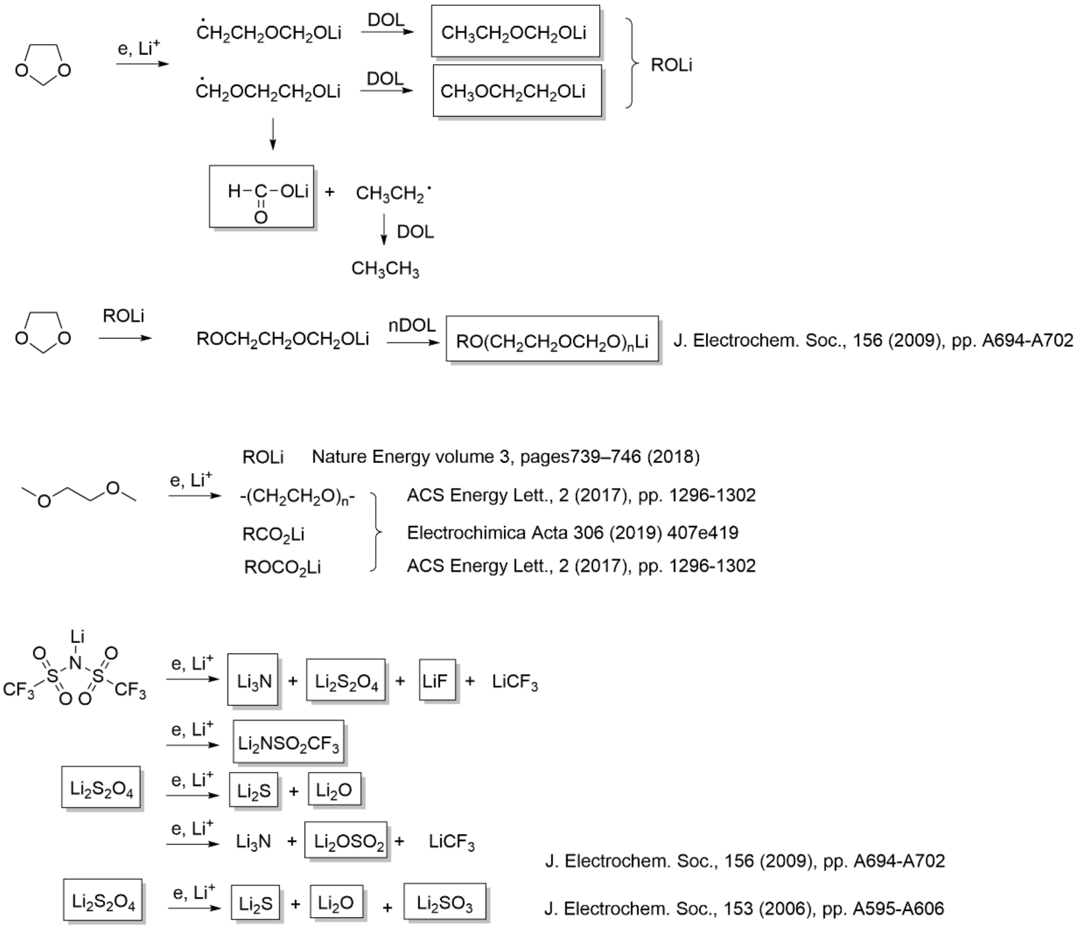

Scheme S1. Formation of the SEI components through the reactions of the electrolyte and solvents in the LiTFSI in DOL/DME electrolyte.[2,8,13–15]

Table S5. Comparison of the electrochemical performance of Li_2DHBQ with that of other lithium-salt based batteries.

Material	Cathode composition	Experimental Capacity, mA g^{-1}	Cycling stability (capacity at the last cycle, current rate)	Refs.
Li2DHBQ	AM/SP/SA = 6:3:1	254 mAh g^{-1}, 100 mA g^{-1}	187 mAh g^{-1} (200th), 500 mA g^{-1}	**This work**
Li_4-*p*-DHBDS	AM/KB/PTFE = 6.5:3:0.5	135 mAh g^{-1}, 9.11 mA g^{-1}	105 mAh g^{-1}(50th), 9.11 mA g^{-1}	[16]
$Li_2PDHBQS$	AM/KB/PTFE = 6:3:1	262 mAh g^{-1}, 50 mA g^{-1}	220 mAh g^{-1}(1500th), 500 mA g^{-1}	[17]
LiOTAP	AM/C65/PVDF = 4.5:50:0.5	385 mAh g^{-1}, 47 mA g^{-1}	200 mAh g^{-1}(100th), 47 mA g^{-1}	[18]
$Li_4C_6O_6$	AM/KB = 8:2	200 mAh g^{-1}, 20 mA g^{-1}	170 mAh g^{-1}(15th), 20 mA g^{-1}	[19]
Li_4-*p*-DHT	AM/KB = 66:33	120 mAh g^{-1}, 25 mA g^{-1}	110 mAh g^{-1}(80th), 25 mA g^{-1}	[20]

References

[1] H. E. Gottlieb, V. Kotlyar, A. Nudelman, *J. Org. Chem.* **1997**, *62*, 7512.
[2] D. Aurbach, E. Pollak, R. Elazari, G. Salitra, C. S. Kelley, J. Affinito, *J. Electrochem. Soc.* **2009**, *156*, A694.
[3] Q. Liu, A. Cresce, M. Schroeder, K. Xu, D. Mu, B. Wu, L. Shi, F. Wu, *Energy Storage Mater.* **2019**, *17*, 366.
[4] H. Bryngelsson, M. Stjerndahl, T. Gustafsson, K. Edström, *J. Power Sources* **2007**, *174*, 970.
[5] H. Jin, H. Liu, H. Cheng, P. Zhang, M. Wang, *J. Electroanal. Chem.* **2020**, *874*, 114484.
[6] A. Lahiri, N. Borisenko, A. Borodin, M. Olschewski, F. Endres, *Phys. Chem. Chem. Phys.* **2016**, *18*, 5630.
[7] D. Zhang, Z. Liu, Y. Wu, S. Ji, Z. Yuan, J. Liu, M. Zhu, *Adv. Sci.* **2022**, *9*, 2104277.
[8] S. Jiao, X. Ren, R. Cao, M. H. Engelhard, Y. Liu, D. Hu, D. Mei, J. Zheng, W. Zhao, Q. Li, N. Liu, B. D. Adams, C. Ma, J. Liu, J.-G. Zhang, W. Xu, *Nat. Energy* **2018**, *3*, 739.
[9] H. Cheng, C. Zhu, M. Lu, Y. Yang, *J. Power Sources* **2007**, *173*, 531.
[10] Y. Pan, G. Wang, B. L. Lucht, *Electrochim. Acta* **2016**, *217*, 269.
[11] S. H. Ju, I.-S. Kang, Y.-S. Lee, W.-K. Shin, S. Kim, K. Shin, D.-W. Kim, *ACS Appl. Mater. Interfaces* **2014**, *6*, 2546.
[12] P. Liu, Y. Rao, H. Wang, X. Li, X. Wang, M. Yu, Y. Li, Z. Yue, F. Wu, S. Fang, *Batteries & Supercaps* **2024**, *7*, e202300353.
[13] H. Yu, J. Zhao, L. Ben, Y. Zhan, Y. Wu, X. Huang, *ACS Energy Lett.* **2017**, *2*, 1296.
[14] C. Zhang, Q. Lan, Y. Liu, J. Wu, H. Shao, H. Zhan, Y. Yang, *Electrochim. Acta* **2019**, *306*, 407.
[15] P. C. Howlett, N. Brack, A. F. Hollenkamp, M. Forsyth, D. R. MacFarlane, *J. Electrochem. Soc.* **2006**, *153*, A595.
[16] A. E. Lakraychi, E. Deunf, K. Fahsi, P. Jimenez, J.-P. Bonnet, F. Djedaini-Pilard, M. Bécuwe, P. Poizot, F. Dolhem, *J. Mater. Chem. A* **2018**, *6*, 19182.
[17] Z. Song, Y. Qian, X. Liu, T. Zhang, Y. Zhu, H. Yu, M. Otani, H. Zhou, *Energy Environ. Sci.* **2014**, *7*, 4077.
[18] A. Slesarenko, I. K. Yakuschenko, V. Ramezankhani, V. Sivasankaran, O. Romanyuk, A. V. Mumyatov, I. Zhidkov, S. Tsarev, E. Z. Kurmaev, A. F. Shestakov, O. V. Yarmolenko, K. J. Stevenson, P. A. Troshin, *J. Power Sources* **2019**, *435*, 226724.
[19] H. Chen, M. Armand, M. Courty, M. Jiang, C. P. Grey, F. Dolhem, J.-M. Tarascon, P. Poizot, *J. Am. Chem. Soc.* **2009**, *131*, 8984.
[20] A green Li–organic battery working as a fuel cell in case of emergency - Energy Environ. Sci. (RSC Publishing) DOI:10.1039/C3EE40878G.
